# Toward fluorescence digital twins: multi-parameter experimental validation of fluorescence Monte Carlo simulations using solid phantoms

**DOI:** 10.1117/1.JBO.30.S3.S34104

**Published:** 2025-05-27

**Authors:** Mayna H. Nguyen, Ethan P. M. LaRochelle, Edwin A. Robledo, Alberto J. Ruiz

**Affiliations:** QUEL Imaging, White River Junction, Vermont, United States

**Keywords:** Monte Carlo simulations, fluorescence, experimental validation, phantoms

## Abstract

**Significance:**

As fluorescence-guided surgery (FGS) gains clinical adoption, robust and experimentally validated computational models for tissue fluorescence are increasingly essential. Although there have been several developments in modeling fluorescence with Monte Carlo simulations, the scope of the experimental validation has been limited in the parameters tested and phantoms used.

**Aim:**

We aim to present and experimentally validate a graphics processing unit (GPU)-accelerated, voxel-based Monte Carlo fluorescence framework capable of modeling varying fluorophore concentrations, optical properties, and complex three-dimensional (3D) geometries.

**Approach:**

A two-step approach (MCX-ExEm) based on Monte Carlo eXtreme was developed for simulating fluorescence. Both commercial reference targets and custom 3D-printed phantoms with well-characterized optical properties were imaged for varying parameters—including absorption, scattering, fluorophore concentrations, and geometries—and compared against simulations.

**Results:**

Strong agreement is observed between simulated and experimental fluorescence across all tested parameters. MCX-ExEm accurately captures nonlinear quenching at high fluorophore concentrations, variations driven by scattering and absorption, intensity scaling with volume, and depth-dependent attenuation and resolution. Minor deviations occur primarily under low-scattering or low-absorption regimes, where optical characterization presents greater uncertainties.

**Conclusions:**

By integrating experimentally validated simulations with a broad range of solid phantoms, this framework establishes a foundation for developing fluorescence digital twins, enabling faster and more systemic testing of fluorescence imaging systems. These findings can help accelerate the design and optimization of FGS and other fluorescence-based biomedical applications.

## 1 Introduction

Monte Carlo modeling software provides a computational method to simulate light transport through tissue. Various Monte Carlo tools have been developed over the last few decades, utilizing statistical methods well-suited for complex geometries commonly observed in biomedical applications, where analytical solutions are not feasible. Monte Carlo simulations can provide precise predictions of light propagation, supporting numerous innovations in medical imaging, diagnostics, and therapeutic interventions. Various Monte Carlo implementations have emerged to meet diverse research and clinical needs, evolving alongside technology advancements. Early central processing unit (CPU)-based tools, such as Monte Carlo for multi-layered media (MCML),^1^ laid the groundwork for tissue modeling, whereas user-friendly platforms such as MCMatlab^2^ offer flexibility and accessibility for researchers. Commercial software, such as TracePro,^3^ integrates Monte Carlo methods with computer-aided design (CAD)-based optical modeling, enabling advanced simulations of light in complex systems. More recently, open-source graphics processing unit (GPU)-accelerated frameworks such as Monte Carlo eXtreme (MCX) and mesh-based Monte Carlo (MMC) have drastically improved computational efficiency, allowing fast simulations of high photon counts in three-dimensional models.^4^^,^^5^

Keijzer et al. and Welch et al. were among the first to demonstrate the use of Monte Carlo simulations for modeling fluorescence. Generally, fluorescence is modeled by running a Monte Carlo simulation for the excitation light, generating a fluorescent source from the first simulation, and running a second Monte Carlo simulation for the emission.^6^^–^^8^ Swartling et al.^9^ describe several approaches that build upon MCML code to speed up modeling fluorescence, including a convolution method and a white Monte Carlo method which scales for the absorption afterward. These methods of simulating fluorescence have been expanded by several groups for applications such as modified fiber optic probe geometries, multilayered geometries, and multiple fluorophores, taking into account the spatial distribution of fluorophores, noncontact measurements, cylindrical diffusing fibers, extracting intrinsic fluorescence, Raman spectroscopy, clinical measurements, and more.^10^^–^^21^ More recent work on fluorescence Monte Carlo simulations utilizes voxel-based approaches and GPUs to accelerate the runtime.^14^^,^^16^^,^^17^

As fluorescence-guided surgery (FGS) continues to gain clinical traction—including recent approvals of targeted fluorescent agents such as Cytalux (pafolacianine) and Lumisight (pegulicianine) for ovarian, lung, and breast cancer^22^^–^^25^—there is a growing need for robust, experimentally validated computational models that can accurately capture the complexity of fluorescence in biological tissues. Despite the expansion of these simulations, the scope of experimental validation has been comparatively limited. For instance, Liu et al.^11^ showed good agreement between simulations and experiments over a range of absorption and scattering coefficients but relied on liquid phantoms containing materials prone to settling. Ong et al.^13^ validated depth sensitivity in two-layer agar phantoms, whereas Pery et al.^12^ and Baran and Foster^14^ validated multilayer and cylindrical diffusing fiber models using agar or liquid phantoms. Vishwanath and Mycek^26^ extended fluorescence Monte Carlo to bi-layered media in a time-resolved context, although certain expansions of the simulations were not experimentally tested. In general, these studies all used liquid- or gel-based phantoms, which have a limited shelf life and derived optical properties from theoretical estimates. There is a need for broader validation of fluorescence Monte Carlo methods using systematically measured optical properties and examining a wide range of fluorophore concentrations and geometries.

Well-characterized optical phantoms are essential for validating fluorescence simulations by providing a consistent reference (“ground truth”) for comparing experimental and simulated outcomes. Tissue-mimicking phantoms, which replicate the absorption and scattering of biological tissue, are particularly valuable for testing clinical and preclinical imaging systems.^27^^–^^29^ Phantom manufacturing methods have been well documented and are commonly developed on an *ad hoc* basis.^29^ Liquid and gelatin phantoms can be challenging to characterize and have limited ability to customize their geometry. Solid epoxy- or resin-based phantoms can be made in customized shapes while often providing a longer shelf life and ease in optical property characterization. The gold standard for measuring optical properties across a wide wavelength range utilizes an integrating sphere to measure the total transmittance and reflectance of a sample for a known thickness and then uses an inverse adding doubling method to estimate the absorption and reduced scattering coefficients.^30^^,^^31^ As this characterization method requires a homogenous material with an even thickness, solid phantoms are ideally suited for this process.

Fluorescence optical phantoms have been demonstrated as a method to characterize the performance of clinical and pre-clinical fluorescence imaging systems.^32^^,^^33^ Recent guidance by both the Food and Drug Administration (FDA) and an international panel of medical physicists suggests the use of fluorescence optical phantoms for imaging system characterization.^28^^,^^34^ These phantoms, in addition to mimicking the optical properties of tissue, contain fluorescent compounds, ideally representative of those used for pre-clinical or clinical use cases.^35^^,^^36^ Although serial dilutions of fluorophores are widely used for *ad hoc* sensitivity evaluations, they often lack appropriate or well-characterized tissue-mimicking optical properties.^28^ In recent years, solid fluorescence phantoms have been demonstrated as a reliable method for producing stable phantoms with consistent properties in applicable geometries.^33^^,^^37^^,^^38^ Furthermore, additive manufacturing [i.e., three-dimensional (3D) printing] techniques allow for complex geometries, providing an opportunity to develop more biologically representative and clinically relevant tools.^37^^–^^39^ Another advantage of utilizing 3D-printed phantoms is the ability to utilize these models in Monte Carlo simulations, thus facilitating the creation of digital twins.

Here, we present a validated, GPU-accelerated two-step Monte Carlo simulation approach that supports voxel-based fluorescence simulations for complex 3D geometries. The simulations were validated against experimental results from increasingly complex phantoms and geometries, spanning a broad range of fluorophore concentrations, absorption and scattering properties, and spatial configurations. To systematically test these conditions, we used both custom-manufactured and commercially available phantoms with well-characterized optical properties. As indocyanine green (ICG) is the most widely used fluorophore in FGS, these phantoms provide a clinically relevant benchmark for validation. These well-characterized physical phantoms form the basis for validating our simulation methods, demonstrating the capability to create digital twins—physical and virtual models with custom geometries and optical properties that can accurately replicate real-world fluorescence behavior. As physical phantom designs are often constrained by fabrication limitations, validated simulations provide a means to extend these reference targets beyond their physical implementations, enabling virtual testing of additional fluorophore concentrations, optical properties, and depth configurations. This integration of physical phantoms with validated, voxel-based Monte Carlo simulations strengthens the framework for digital twins in fluorescence imaging, supporting applications in imaging system characterization and fluorescence-guided surgical tool development while providing a robust framework for iterative design. Ultimately, the development of validated digital twins offers the potential to streamline innovation in fluorescence imaging, accelerate device development, and reduce *in vivo* experimentation.

## 2 Methods

### 2.1 Two-Step Monte Carlo Fluorescence Simulation Overview (MCX-ExEm)

**Fig. 1:**
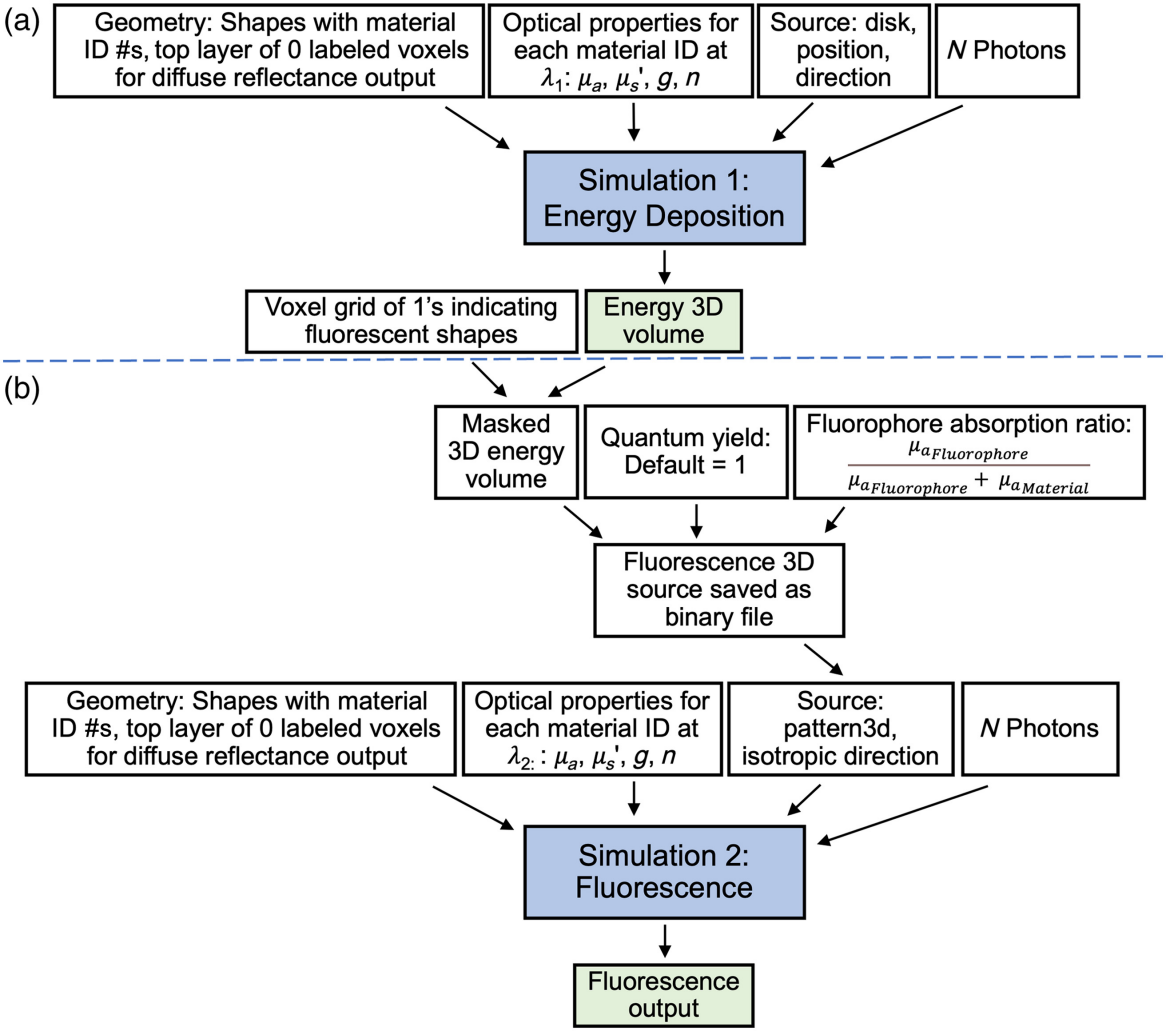
Flowchart outlining the use of MCX in a two-step Monte Carlo simulation process for modeling fluorescence (MCX-ExEm). (a) Step 1 computes photon propagation and energy deposition at the excitation wavelength. (b) Step 2 uses that deposited energy as a volumetric source for emission.

MCX^4^ is an open-source, GPU-accelerated light transport simulator that has been extensively validated for modeling photon propagation in 3D turbid media. Its robust framework is well-suited for extending Monte Carlo methods into fluorescence applications, without making any modifications to the base code. The simulations presented in this work are generated using MCX v2024.1 operated on a Thelio Mira desktop workstation (System76, Denver, Colorado, United States) running Ubuntu 20.04. The computer specifications include a 5.0-GHz Intel 11th Gen i7 processor (8 cores, 16 threads), 64 GB DDR4 RAM, and an NVIDIA GeForce RTX 3090 GPU (24 GB VRAM, 10,496 CUDA cores) running CUDA version 12.2 (NVIDIA-SMI 535.104.05). MCX was installed using a Docker container to consistency and portability of the software, and the installation received an MCX GPU benchmark score of 244,744 photons/ms. Here, we present a two-step simulation approach, which we refer to as MCX-ExEm (Fig. 1) that leverages MCX’s capabilities to sequentially simulate the excitation and emission processes central to fluorescence. By incorporating experimentally measured optical properties and fluorophore parameters, this method enables precise simulations of fluorescence propagation in turbid media. Specifically, step 1 models how excitation photons propagate and deposit energy, whereas step 2 uses this deposited energy—restricted to the fluorophore-containing regions—as the source for emission. The resulting fluorescence distributions provide outputs that mimic images captured by fluorescence systems, enabling direct validation against tissue-mimicking phantoms and facilitating the development of fluorescence imaging digital twins.

Each simulation step took between 1 and 10 s to run with $10^{7}$ photons, with some additional data aggregation and processing steps in between (seconds to <3  min for the most complex geometry). As shown in Fig. S1 and Table S1 in the Supplementary Material, different input photon numbers were tested ($10^{3}$ to $10^{9}$), and the scaled region-of-interest (ROI) value converged to <0.1% error of the $10^{9}$ ROI value when using $10^{7}$ photons for a simple concentration well, which is representative of most geometries in this study. The data in Fig. S2 and Table S2 in the Supplementary Material show similar results for the more complex depth resolution geometry (see Sec. 2.4.6), with results starting to converge at $10^{7}$ photons. Thus, all simulations were run with $10^{7}$ photons, as this shows good convergence and quick simulation times.

#### 2.1.1 Simulation 1: energy deposition and fluorophore excitation

In the first step of MCX-ExEm [Fig. 1(a)], the geometries, material optical properties at the excitation wavelength, and source properties are defined in an input JavaScript Object Notation (JSON) file. In MCX, the geometries are defined by shapes that each have a tag number, which assigns the voxels in the shape a material ID and the respective defined optical properties. The background material optical properties are also set, with no absorption or scattering and a refractive index value of n=1. For all simulations described below, the source is defined as a collimated, flat-top disk with a specified beam diameter and direction. Fluorescence is recorded via diffuse reflectance, which is defined by MCX as the photon area density on the surface of an object. The diffuse reflectance is recorded by adding a layer of 0 labeled voxels to the surface to be imaged in the geometry set in the JSON file. The fluorophore absorption coefficient is calculated (see Sec. 2.2.1) for the excitation wavelength ($\lambda_{1}$) and is added to the absorption coefficient of the geometries that contain the fluorophore. This JSON file is then used as an input to MCX with input flags to specify Fresnel reflection boundaries to capture internal reflections. At this step, $10^{7}$ is the typical number of input photons used in the present work, and normalization is enabled so that the total energy launched is 1 J for simulation comparisons. The output of this simulation is the 3D energy deposition map that is utilized as an input to the fluorescence emission simulation.

#### 2.1.2 Simulation 2: fluorescence emission and propagation

In the second step of MCX-ExEm [Fig. 1(b)], the same geometries are used, but the material optical properties are updated to be at the emission wavelength ($\lambda_{2}$). The fluorophore absorption coefficient is recalculated and added to the same fluorophore-containing geometries. The source is updated to a 3D pattern source based on the energy deposition map from the first simulation and is then masked so that only the voxels containing fluorophore are retained. This is done by defining a 3D array of 0’s, voxelizing the entire geometry, and then defining only the fluorescent voxels as 1’s. This voxel grid is multiplied by the energy deposition map and the fluorophore absorption ratio

$$\text{Fluorophore Absorption Ratio}=\frac{\mu_{a\mathrm{Fluorophore}}}{\mu_{a\mathrm{Fluorophore}}+\mu_{a\mathrm{Material}}},$$

which represents the fraction of local absorption attributable to the fluorophore, determining how much of the deposited energy is converted into fluorescence. This product is further multiplied by the fluorophore’s quantum yield. As only one fluorophore, ICG, is used in this work, the quantum efficiency is set to 1 for simplicity.

The resulting product is saved as a binary file and serves as the 3D pattern source for the second simulation. When using the 3D pattern source in MCX, the N input photons are uniformly distributed into each voxel of the fluorescent volume. The photons are then weighted by the values set in the binary file and are set to isotropically launch. MCX is then run with this second JSON file with the defined number of photons ($\geq 10^{7}$). Input arguments are used to specify Fresnel reflection boundaries to capture internal reflections, and normalization is disabled so that the total energy is derived exclusively from the modified deposited energy, allowing for accurate comparisons among simulations. The output of the second step is the diffuse reflectance on the imaging surface, which serves as the basis of the fluorescence imaging digital twin output and is utilized for simulation visualization and ROI analysis.

### 2.2 Fluorescence and Optical Property Determination for Simulation

Accurate modeling of fluorescence in turbid media requires reliable estimates of both the fluorophore’s optical properties and those of the surrounding bulk material. The following subsections detail the procedures for determining the extinction coefficient of ICG through absorbance measurements (Sec. 2.2.1) and for characterizing the absorption and scattering coefficients of the 3D-printed phantom materials (Sec. 2.2.2).

#### 2.2.1 ICG absorption coefficient calculation

To inform the excitation and emission simulations, the extinction coefficients of ICG at 785 and 820 nm were determined by measuring the absorbance spectra of liquid ICG samples. A stock solution of 10,000 nM ICG in dimethyl sulfoxide (DMSO) (Sigma-Aldrich, St. Louis, Missouri, United States) was serially diluted to concentrations of 3000, 1000, 300, 100, and 30 nM, and a cuvette of each concentration was prepared for analysis on a Duetta spectrofluorometer (Horiba, Kyoto, Japan). The absorbance spectra of each cuvette were measured with an integration time of 0.1 s, and the baseline from the measurement of a control cuvette containing only DMSO was subtracted. Figure S3 in the Supplementary Material presents the resulting spectra.

The extinction coefficient was determined from Beer’s law

A=εcl,

where A is the absorbance, ε is the molar extinction coefficient ($M^{-1}\;{\mathrm{cm}}^{-1}$), c is the concentration (M), and l is the path length of the cuvette (1 cm = 10 mm). Using Beer’s law, the extinction coefficient was determined by calculating the average slope of the absorbance versus concentration data. As shown in Fig. S1(b) in the Supplementary Material, from these fits, we estimate the extinction coefficients to be $∼17,500\;M^{-1}\;{\mathrm{mm}}^{-1}$ at 785 nm and $8500\;M^{-1}\;{\mathrm{mm}}^{-1}$ at 820 nm.

To calculate the ICG absorption coefficient $\mu_{a\mathrm{ICG}}$, the following equation was used:

$$\mu_{a\mathrm{ICG}}=\frac{2.303A}{l}=2.303\varepsilon c,$$

where the factor 2.303 converts between base-10 and base-*e* logarithms (absorbance is measured in base-10, whereas optical properties in light transport equations typically use base-*e*). Although the simulations and experiments in this study focus on ICG, this method can be applied to any fluorophore by measuring its absorbance and determining its molar extinction coefficient at the excitation and emission wavelengths.

#### 2.2.2 Optical property measurements

Optical property measurements were performed on 50×50  mm samples, each printed at thicknesses ranging from 2 to 5 mm according to the material type. These materials correspond to the formulations used in the various phantoms examined in subsequent sections. Total transmittance and total reflectance values were measured with a Lambda 1050+ Double-Beam Spectrophotometer (PerkinElmer, Waltham, Massachusetts, United States) with the 150-mm integrating sphere accessory. The measured total transmittance and reflectance values were then analyzed via an inverse adding doubling algorithm written by Prahl et al.^31^^,^^40^ (v3.12.1) to determine the absorption coefficient $\mu_{a}$ and reduced scattering coefficient $\mu_{s}^{\prime }$ over the 500 to 1000 nm range. The refractive index of 1.5 was informed by single-wavelength measurements of the base material at 660 nm, whereas the anisotropy factor of g=0.85 was estimated based on reported values for titanium dioxide (${\mathrm{TiO}}_{2}$).^41^^–^^43^ It is worth noting that for samples thick enough to exhibit diffuse-regime behavior—such as the 2 to 5 mm samples used here—the impact of these assumptions on the extracted optical properties is reduced, as light transport is increasingly dominated by multiple scattering events. The scattering coefficient was calculated from the reduced scattering coefficient using

$$\mu_{s}=\frac{\mu_{s}^{\prime }}{\left(1-g\right)}.$$

### 2.3 Fluorescence Imaging

All fluorescence imaging of phantoms was performed on a custom fluorescence imaging system (QUEL Imaging, White River Junction, Vermont, United States) that utilizes a 785 nm laser excitation, 800 nm long-pass filter, ∼35 mm focal length lens, ∼200 mm working distance, and a cooled complementary metal oxide semiconductor (CMOS) camera. The fluorescence emission data were captured as uncompressed 16-bit monochrome images. Image processing was done utilizing ImageJ and Python code to extract ROI values and plot profiles as described in each respective section.

### 2.4 Simulations and Experiments

To validate the MCX-ExEm fluorescence approach under diverse experimental conditions, we systematically increased the complexity of both simulations and measurements. We began with a simple cuvette model to confirm concentration-dependent fluorescence behavior, including nonlinear effects. Next, we examined how fluorescence scales with varying bulk scattering, absorption, and volume, before moving on to standardized reference targets that incorporate both concentration and depth variations. Finally, we evaluated a channel-based phantom geometry to assess depth resolution and confirm the robustness of the simulation framework under more complex spatial configurations.

#### 2.4.1 Cuvette with varying ICG concentrations

##### Experiment

Six cuvettes with ICG in DMSO were prepared at concentrations of 30, 100, 300, 1000, 3000, and 10,000 nM. Fluorescence emission spectra ($\lambda_{\mathrm{excitation}}=785\;\mathrm{nm}$) were measured with a Duetta spectrofluorometer (Horiba, Kyoto, Japan). Data were collected with a 2 s integration time for concentrations 30 to 1000 nM and a 1 s integration time for the 3000 and 10,000 nM concentrations. The resulting spectra were scaled appropriately for integration time, and the intensity values at 820 nm were extracted for comparison with simulations.

##### Simulation

To replicate the experimental conditions, the liquid region of the cuvette was modeled in MCX as a 10×10×20  mm box (grid size = 0.1 mm), excluding the physical walls of the cuvette. The source was a 5 mm diameter disk beam centered and oriented toward the cuvette, with the detector placed on a lateral surface at a 90-deg angle from the source. Six simulations were run to replicate the experimental conditions. The cuvette volume was assumed to have the optical properties of water,^44^ assuming no scattering ($\mu_{a}=0.002\;{\mathrm{mm}}^{-1}$, $\mu_{s}=0$, g=0, n=1.33), and an ICG absorption coefficient (see Sec. 2.2.1) that varied by concentration (30, 100, 300, 1000, 3000, and 10,000 nM) was calculated for the excitation and emission simulations. Both excitation and emission simulations used $10^{7}$ photons and a 0.1 mm grid length.

##### Analysis

For the experiment, the absolute intensities were taken from the spectrofluorometer. These values are then compared with the sum of the fluorescence output (i.e., diffuse reflectance) from the simulations. The experiment and simulation datasets were normalized to the respective max value.

The normalized absolute error is calculated as

$$\mathrm{Normalized}\;\mathrm{Absolute}\;\mathrm{Error}\;=\mathrm{abs}(\frac{\mathrm{simulation}\;\mathrm{fluorescence}\;\mathrm{intensity}}{\max(\mathrm{simulation}\;\mathrm{fluorescence}\;\mathrm{intensity})}\;-\frac{\mathrm{experiment}\;\mathrm{fluorescence}\;\mathrm{intensity}}{\max(\mathrm{experiment}\;\mathrm{fluorescence}\;\mathrm{intensity})}).$$

All errors are reported in the Supplementary Material.

#### 2.4.2 Bulk scattering and bulk absorption

##### Experiment

Solid phantoms were fabricated using a 3D printing process adapted from Ruiz et al.^37^ with nigrosin as the absorber, ${\mathrm{TiO}}_{2}$ as the scatterer, and ICG as the fluorophore (300 nM). Eight proprietary formulations were developed to span a range of reduced scattering ($\mu_{s}^{\prime }\approx 0$ to ${\mathrm{mm}}^{-1}$) and absorption ($\mu_{a}\approx 0.02$ to $0.1\;{\mathrm{mm}}^{-1}$) coefficients. Cylindrical phantoms (10 mm diameter ×20  mm length) were 3D-printed for imaging, and 50×50  mm cards (4 and 6 mm thick) were co-manufactured for optical property measurements. After post-curing using a 405-nm illumination, the phantoms were placed into custom absorbing holders (3D-printed black PLA) and imaged individually (see Sec. 2.3 for imaging details) using consistent settings across all samples.

##### Simulation

To characterize the effects of varying bulk scattering and absorption on fluorescence, eight simulation sets were performed using the MCX-ExEm method with the measured optical properties [see Sec. 3.2 and Figs. 3(a) and 3(b)]. The refractive index and anisotropy were assumed to be 1.5 and 0.85, respectively (see Sec. 2.2.2). As the measured optical properties included ICG in the phantoms (300 nM), the ICG absorption coefficient was not added to but included with the overall absorption coefficient. However, the calculated absorption coefficient for 300 nM ICG was still used for the fluorophore absorption ratio. The geometry for each simulation was a 10 mm diameter × 20 mm length cylinder of fluorescent material with varying optical properties, surrounded by a 20×20×20  mm absorbing cube ($\mu_{a}=1000\;{\mathrm{mm}}^{-1}$, $\mu_{s}=0\;{\mathrm{mm}}^{-1}$) to mimic the experimental phantoms. A 10 mm diameter disk source was centered and directed onto the fluorescent cylinder. The MCX-ExEm simulation was run with $10^{7}$ photons and a 0.1 mm grid length for both steps.

##### Analysis

The mean ROI value is calculated by measuring the average pixel count in the central circular region that is half the diameter (5 mm) of the circular imaging surface. The ROI was chosen to replicate experimental guidance as described in Pogue et al.,^28^ which uses the half-diameter interior region to avoid effects of blurring at the edges of the well walls. This was done for both the experimental images and simulations. The ROIs were then normalized to the max value of the experimental values and simulation values. The normalized absolute error is calculated as

$$\mathrm{Normalized}\;\mathrm{Absolute}\;\mathrm{Error}=\mathrm{abs}\;(\frac{\mathrm{simulation}\;\mathrm{mean}\;\mathrm{ROI}}{\max(\mathrm{simulation}\;\mathrm{mean}\;\mathrm{ROI})}-\frac{\mathrm{experiment}\;\mathrm{mean}\;\mathrm{ROI}}{\max(\mathrm{experiment}\;\mathrm{mean}\;\mathrm{ROI})}).$$

All errors are reported in the Supplementary Material.

#### 2.4.3 Varying volumes

##### Experiment

Six cylindrical phantoms of increasing diameter (2 to 20 mm, see Table S3 in the Supplementary Material) were manufactured alongside 50×50  mm optical samples with the same process described in Sec. 2.4.2. The cylindrical phantoms were placed into a custom highly absorbing mold, and the entire assembly was imaged as a single unit (see Sec. 2.3 for imaging details).

##### Simulation

To simulate the fluorescence of cylinders of different dimensions, a 30×30×20  mm grid (grid length = 0.1 mm) of absorbing material ($\mu_{a}=1000$, $\mu_{s}=0$) was defined first, and then the cylinders with the diameters, heights, and volumes shown in Table S3 in the Supplementary Material were defined on top of the grid. Each cylinder was simulated separately. The base optical properties were measured: at 785 nm, $\mu_{a}=0.064\;{\mathrm{mm}}^{-1}$, $\mu_{s}^{\prime }=1.275\;{\mathrm{mm}}^{-1}$; at 820 nm, $\mu_{a}=0.061\;{\mathrm{mm}}^{-1}$, $\mu_{s}^{\prime }=1.257\;{\mathrm{mm}}^{-1}$. The same estimates for anisotropy and refractive index were used for both wavelengths (g=0.85, n=1.5, see Sec. 2.2.2). A $\mu_{a}$ for a concentration of 300 nM ICG was used for the fluorophore and added to the overall absorption coefficient. A 20 mm diameter disk source was placed at the top surface and directed toward the cylinder with $10^{7}$ photons used to emulate a wide-field source. The simulation was run using the MCX-ExEm process described above, but the number of photons used in the second simulation was adjusted so that $10^{7}$ photons were multiplied by a scaling factor of

$$\frac{\mathrm{Cylinder}(\mathrm{diameter})\;\mathrm{volume}}{\mathrm{Cylinder}\;(20\;\mathrm{mm}\;\mathrm{diameter})\;\mathrm{volume}}$$

to account for scaling differences when using a 3D pattern source. In short, when using the 3D pattern source in MCX, N photons are uniformly distributed into each voxel of the fluorescent region. This means that smaller volumes with less voxels will have more starting photons per voxel. To simulate fluorescence across varying geometries, the source should be an equal number of starting photons per voxel multiplied by the absorbed energy values in the binary file (see Fig. 1), where the absorbed energy is the varying factor. Therefore, the number of photons N must be scaled with volume to ensure the same number of photons per voxel which enables comparisons of simulations.

##### Analysis

The mean ROI value is calculated by measuring the average pixel count in the central circular region that is half the diameter of each cylinder. The ROI was chosen to replicate experimental guidance as described in Pogue et al.,^28^ which uses the half-diameter interior region to avoid the effects of blurring at the edges of the well walls. This was done for both the experimental images and simulations. The ROIs were then normalized to the max value of the experimental values and simulation values. The normalized absolute error is calculated as described in Sec. 2.4.2.

#### 2.4.4 Concentration sensitivity target

##### Experiment

An ICG-equivalent concentration sensitivity target (QUEL Imaging, White River Junction, Vermont, United States) was imaged as per Sec. 2.3. In brief, the target uses tissue-equivalent optical formulations in nine wells with varying ICG-equivalent concentrations (1 to 1000 nM alongside a control well) and serves as a shelf-stable fluorescence signal sensitivity target over a wide range of fluorescence signals.

##### Simulation

To simulate the ICG concentration sensitivity target, nine separate MCX-ExEm simulation sets were run for each of the ICG concentrations (1000, 300, 100, 60, 30, 10, 3, 1, and 0 nM) and with the measured phantom optical properties. The simulation geometry was a 10 mm diameter × 20 mm length cylinder with assigned optical properties of $\mu_{a}=0.028\;{\mathrm{mm}}^{-1}$ (785 nm), $0.026\;{\mathrm{mm}}^{-1}$ (820 nm) and $\mu_{s}^{\prime }=0.424\;{\mathrm{mm}}^{-1}$ (785 nm), $0.411\;{\mathrm{mm}}^{-1}$ (820 nm). The assumed refractive index and anisotropy are 1.5 and 0.85, respectively (see Sec. 2.2.2). The cylindrical well is surrounded by 20×20×20  mm cube of absorbing material ($\mu_{a}=1000\;{\mathrm{mm}}^{-1}$, $\mu_{s}^{\prime }=0\;{\mathrm{mm}}^{-1}$, g=0, n=1.5). A 10 mm diameter disk source was centered and directed at the circular imaging surface of each cylinder geometry. The simulations were run using 10^7^ starting photons and a grid length of 0.1 mm.

##### Analysis

The mean ROI value is calculated by measuring the average pixel count in the central circular region that is half the diameter (5 mm) of the circular imaging surface. The ROI was chosen to replicate experimental guidance as described in Pogue et al.,^28^ which uses the half-diameter interior region to avoid the effects of blurring at the edges of the well walls. This was done for both the experimental images and simulations. The ROIs were then normalized to the max value of the experimental values and simulation values. The normalized absolute error is calculated as described in Sec. 2.4.2.

#### 2.4.5 Depth sensitivity target

##### Experiment

An ICG-equivalent depth sensitivity target (QUEL Imaging, White River Junction, Vermont, United States) was imaged as per Sec. 2.3. In brief, the target characterizes how fluorescence signal changes at varying depths within overlaying non-fluorescent material (0.5 to 6 mm depth) for 1000 nM ICG wells alongside a control at a 6 mm depth.

##### Simulation

To simulate the ICG depth sensitivity target, nine separate MCX-ExEm fluorescence simulation sets were run, each with a different thickness of a non-fluorescent scattering top layer (depths = 0.5, 1, 1.5, 2, 3, 4, 5, and 6 mm). The geometry was a 10 mm diameter × (20 − depth layer height) mm cylinder on the bottom, a 10 mm diameter × depth layer height cylinder on top, a surrounding 20×20×20  mm absorbing cube, and a grid length of 0.1 mm. The top layer is a non-fluorescent scattering layer and has optical properties $\mu_{a}=0.011\;{\mathrm{mm}}^{-1}$ (785 nm), 0.0112 mm^−1^ (820 nm) and $\mu_{s}^{\prime }=1.6\;{\mathrm{mm}}^{-1}$ (785 nm), $1.399\;{\mathrm{mm}}^{-1}$ (820 nm). The assumed refractive index and anisotropy are 1.5 and 0.85, respectively (see Sec. 2.2.2). The bottom layer and surrounding absorbing cube had the same optical properties as those defined in Sec. 2.4.4. The ICG concentration for each bottom layer was 1000 nM. The source is a 10 mm diameter disk source centered and directed at the circular imaging surface of each cylinder geometry with $10^{7}$ starting photons.

##### Analysis

The mean ROI value is calculated by measuring the average pixel count in the central circular region that is half the diameter (5 mm) of the circular imaging surface. The ROI was chosen to replicate experimental guidance as described in Pogue et al.,^28^ which uses the half-diameter interior region to avoid the effects of blurring at the edges of the well walls. This was done for both the experimental images and simulations. The ROIs were then normalized to the max value of the experimental values and simulation values. The normalized absolute error is calculated as described in Sec. 2.4.2.

#### 2.4.6 Depth resolution target (FluoFlow® Phantom)

##### Experiment

A FluoFlow® phantom (QUEL Imaging, White River Junction, Vermont, United States) was 3D-printed and characterized as per Sec. 2.2.2 using 3 and 4 mm co-manufactured reference samples. The phantom channel (1.0 mm diameter, varying depth 1 to 6 mm) was filled using a 1000 nM ICG solution in distilled water via the Luer lock connector and imaged as described in Sec. 2.3.

##### Simulation

To simulate the FluoFlow® phantom geometry, the base material was modeled as a 40×30×14  mm grid with measured optical properties [$\mu_{a}=0.0043\;{\mathrm{mm}}^{-1}$ (785 nm), $0.0051\;{\mathrm{mm}}^{-1}$ (820 nm), $\mu_{s}^{\prime }=1.3232\;{\mathrm{mm}}^{-1}$ (785 nm), $1.1376\;{\mathrm{mm}}^{-1}$ (820 nm), g=0.85, n=1.5] (see Sec. 2.2.2). A series of three cylinders, each 1.0 mm in diameter, were embedded in the material to represent the flow channel. The first cylinder spanned x=0  mm to x=5  mm at a constant depth of z=1  mm + channel radius. The second cylinder was angled from x=5  mm to x=35  mm, transitioning in depth from z=1  mm + channel radius to z=6  mm + channel radius. The third cylinder spanned from x=35  mm to x=40  mm, with a constant depth of z=6  mm + channel radius. The cylinders were assigned optical properties corresponding to 1000 nM ICG in water^44^ ($\mu_{a}=0.002\;{\mathrm{mm}}^{-1}$, $\mu_{s}^{\prime }=0\;{\mathrm{mm}}^{-1}$, g=0, n=1.33), assuming no scattering. A 40 mm diameter disk source was centered and positioned on top of the grid at (x=20  mm, y=15  mm, and z=0  mm). A grid length of 0.1 mm and $10^{7}$ photons were used for both excitation and emission steps. For the emission simulation, the voxelized energy-deposition map from the excitation simulation was masked such that only the cylinder regions were retained (assigned as 1), whereas all non-channel voxels were set to 0.

##### Analysis

To assess how fluorescence intensity varies with depth and x-position, the line profile intensity at y=15  mm was measured along the x-axis (0 to 40 mm), min–max normalized, for both the experimental fluorescence image and the simulated diffuse reflectance. A Savitzky–Golay filter was used for smoothing both the simulated image and experimental data. The data were then plotted so that the x-positions are from 0 to 40 mm. For both experimental and simulated data, an exponential curve was fitted between x=5  mm and x=35  mm, corresponding to where the depth ranges from 1 to 6 mm. The normalized absolute error is calculated as described in Sec. 2.4.1.

To quantify the lateral spread of fluorescence, the simulated and experimental images were each min–max normalized and resized to be the same dimensions. Orthogonal line profiles were extracted along 100 uniformly spaced x-positions, smoothed with a Savitzky–Golay filter, and analyzed to determine the full width at half maximum (FWHM) of each profile. The resulting FWHM values were plotted as a function of the x-position.

## 3 Results

### 3.1 Cuvette with Varying ICG Concentrations

**Fig. 2:**
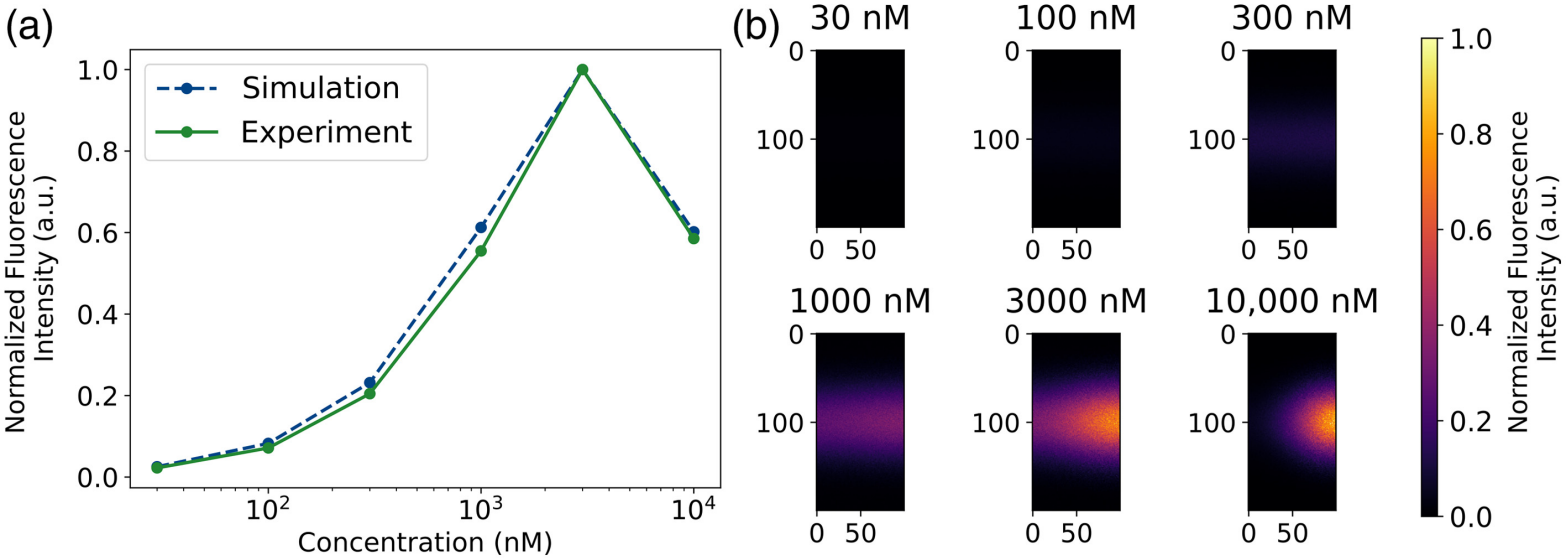
Results of the cuvette experiments and corresponding MCX-ExEm simulations under varying ICG concentrations. (a) Normalized summed fluorescence intensities ($\lambda_{\mathrm{excitation}}=785\;\mathrm{nm}$, $\lambda_{\mathrm{emission}}=820\;\mathrm{nm}$) detected at 90 deg from simulations and experimental data. (b) Simulated image data of a cuvette with varying ICG concentrations (30 to 10,000 nM). Absolute fluorescence intensities of the detector area are summed and normalized to the respective max value.

The results of the varying ICG concentration cuvette experiments and corresponding simulations are shown in Fig. 2. Figure 2(a) shows a strong correlation between simulated and experimental data, with a maximum normalized absolute error of 0.07. Both simulation and experiment exhibit an increase in fluorescence intensity with ICG concentration up to 3000 nM, followed by a decline at 10,000 nM, suggesting fluorescence quenching at higher concentrations. This fluorescence quenching is most likely caused by first-order inner filter effects,^45^ which can be visualized in the simulated fluorescence images shown in Fig. 2(b). The brightest fluorescence is observed at 3000 nM, whereas the intensity diminishes at 10,000 nM.

### 3.2 Bulk Scattering and Bulk Absorption

**Fig. 3:**
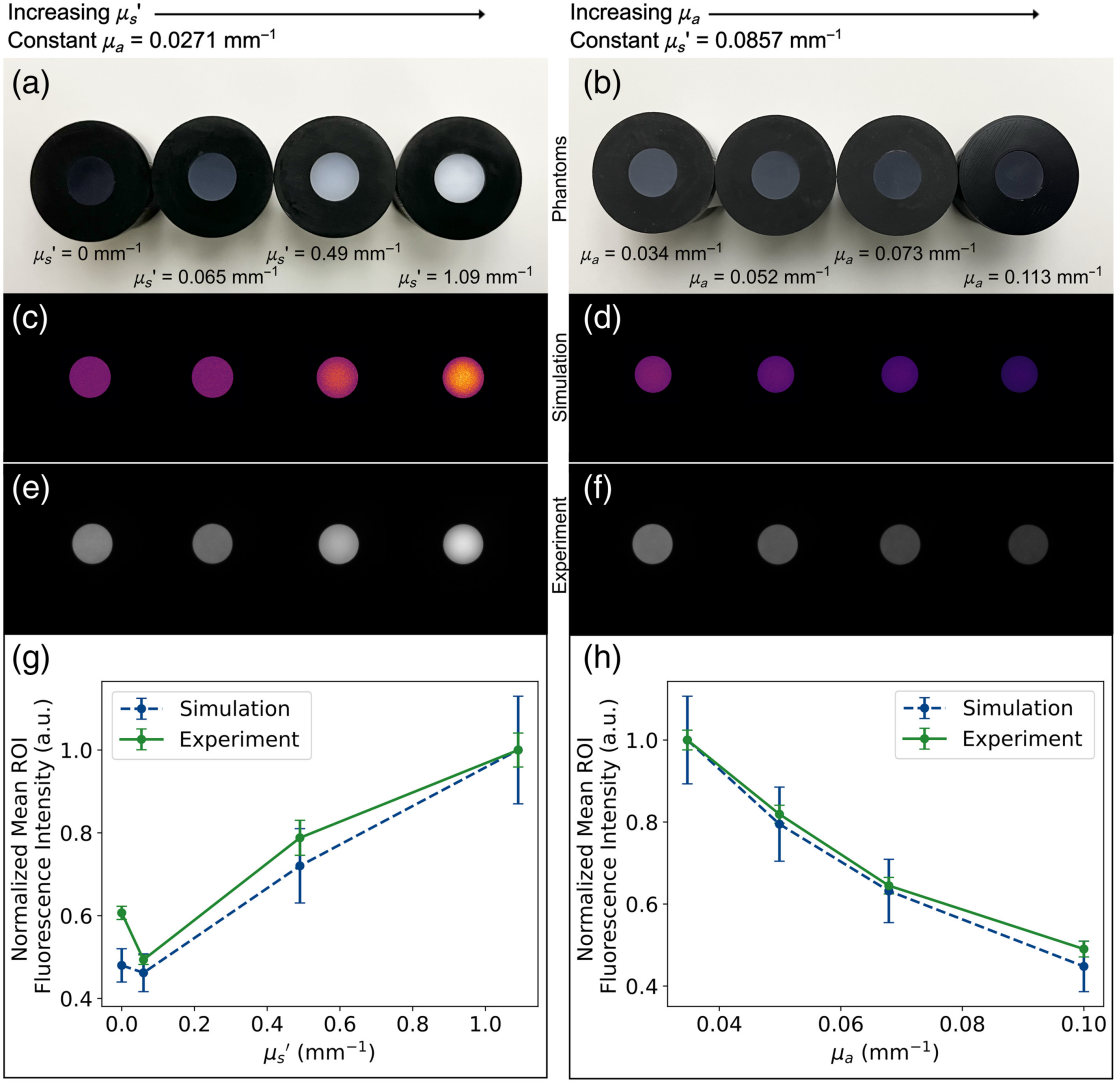
Results of the varying bulk scattering and absorption simulations and experiments. (a) and (b) White light images of the tuned optical property fluorescent phantoms with 300 nM ICG, with (c) and (d) corresponding MCX-ExEm simulated fluorescence images and (e) and (f) experimental fluorescence images. (g) Plot of normalized mean ROI fluorescence intensities versus reduced scattering coefficients for MCX-ExEm simulations and experiments. (h) Plot of normalized mean ROI fluorescence intensities versus absorption coefficients for MCX-ExEm simulations and experiments. Half-diameter ROIs are taken from the simulated and experimental images and are normalized to the respective maximum value. Error bars represent the normalized standard deviation of each respective ROI (simulation and experiment).

A total of eight phantoms were manufactured, measured for optical properties, imaged, and simulated; the results are shown in Fig. 3. The manufactured phantoms and measured optical properties at 785 nm are shown in the figure for varying reduced scattering [Fig. 3(a)] and varying absorption [Fig. 3(b)], with corresponding properties at 820 nm of $\mu_{s}^{\prime }=0,0.063$, 0.476, and $1.050\;{\mathrm{mm}}^{-1}$ and $\mu_{a}=0.0348$, 0.0499, 0.0680, and $0.102\;{\mathrm{mm}}^{-1}$, respectively. As the phantoms for the varying scattering set were manufactured using the same amount of absorber, the absorption coefficient was averaged across samples and set to $\mu_{a}=0.0271\;{\mathrm{mm}}^{-1}$ (785 nm) and $\mu_{a}=0.0279\;{\mathrm{mm}}^{-1}$ (820 nm). Similarly, for the varying absorption set, the reduced scattering coefficient was averaged across samples and set to $\mu_{s}^{\prime }=0.0857\;{\mathrm{mm}}^{-1}$ (785 nm) and $\mu_{s}^{\prime }=0.0826\;{\mathrm{mm}}^{-1}$ (820 nm). These optical properties were then used in the simulations, with the resulting simulated images shown in Figs. 3(c) and 3(d). These simulated images resemble the corresponding experimental fluorescence images shown in Figs. 3(e) and 3(f).

To generate the plots in Figs. 3(g) and 3(h), 5 mm ROIs (half the diameter of the imaging surface)^28^ were extracted from both simulated and experimental fluorescence images and normalized to their respective maxima. These data show that the normalized fluorescence intensities from the simulations closely follow the experimental trends. Figure 3(g) shows a distinctive inflection point where increasing scattering begins to result in higher fluorescence, whereas Fig. 3(h) shows a decrease in fluorescence with increasing absorption. The largest discrepancy occurs for the lowest scattering sample ($\mu_{s}^{\prime }=0$), where the experimental fluorescence is higher than the simulated value (normalized absolute error = 0.1268). This mismatch is likely due to the limitations in our optical characterization methods, as the base material potentially has some scattering and lower scattering materials may be more sensitive to inaccuracies in measured optical properties, affecting both the simulation inputs and experimental signal propagation. The maximum normalized absolute error for the varying absorption values was 0.042, which is well within the normalized standard deviation values.

### 3.3 Varying Volumes

**Fig. 4:**
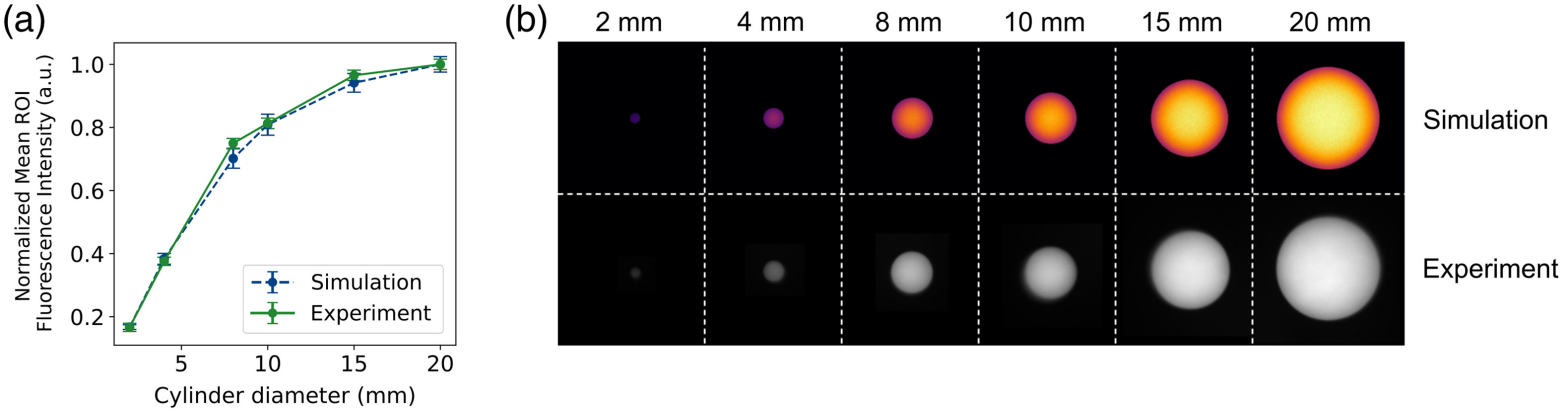
Results of the varying volumes simulations and experiments. (a) Normalized mean ROI (half diameter) fluorescence intensity values for varying cylinder diameters (2, 4, 8, 10, 15, and 20 mm) from (b) MCX-ExEm simulation images (top row) and experimental images (bottom row). Half-diameter ROIs are taken from each cylinder and ROI intensities are normalized to the maximum value of the simulation or experiment values, respectively. Error bars represent the normalized standard deviation of each respective ROI (simulation and experiment).

Figure 4 compares MCX-ExEm simulations and experimental measurements of fluorescence intensity for cylindrical phantoms with varying diameters (2 to 20 mm). Both simulation and experimental results exhibit increasing fluorescence intensity with larger cylinder diameters [Fig. 4(a)]. The fluorescence increases rapidly for smaller diameters and subsequently plateaus as the diameter gets larger. The simulations closely match the experimental data, with a maximum normalized absolute error of 0.024 and as shown by the overlapping error bars. Figure 4(b) shows similar spatial distributions for simulated and experimental images, with larger cylinders exhibiting higher fluorescence intensity.

### 3.4 Concentration Sensitivity Target

**Fig. 5:**
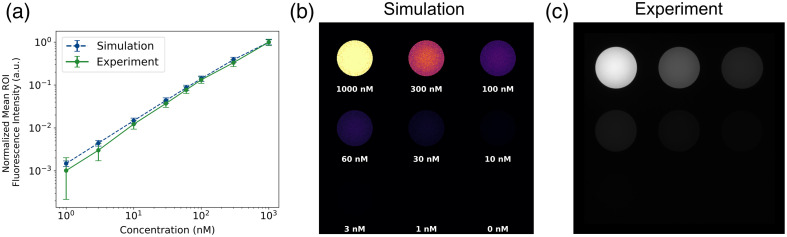
Results of the concentration sensitivity target simulations and experiment. (a) Normalized mean ROI fluorescence intensity versus concentration (1, 3, 10, 60, 100, 300, and 1000 nM) on a log–log scale for MCX-ExEm simulations and experiments corresponding to the (b) MCX-ExEm simulation images and (c) experimental fluorescence image of a QUEL Imaging ICG-equivalent concentration sensitivity target. A 5 mm ROI (half-diameter) was taken from each circular well and normalized to the max value of the simulation or experiment values, respectively. The experiment values subtract the control well baseline before normalization (see the Supplementary Material). Error bars represent the normalized standard deviation of each respective ROI (simulation and experiment).

Simulations and fluorescence image data are compared for a QUEL Imaging ICG-equivalent concentration sensitivity target in Fig. 5. The normalized ROIs [Fig. 5(a)] show strong agreement between simulation and experimental data, following a linear trend on a log–log scale. The experimental intensity values are slightly lower than the simulation values, with a maximum normalized absolute error of 0.058. This error falls within the normalized standard deviations, as shown through the overlapping error bars, indicating no statistically significant differences. Figures 5(b) and 5(c) show the fluorescence images for the simulation and experiment, respectively, with the top left well representing the highest concentration. The fluorescence remains visible down to ∼30  nM in both simulated and experimental images, and then the signal diminishes at lower concentrations. Each image was displayed using a linear look-up table to ensure comparable visualization of the data.

### 3.5 Depth Sensitivity Target

**Fig. 6:**
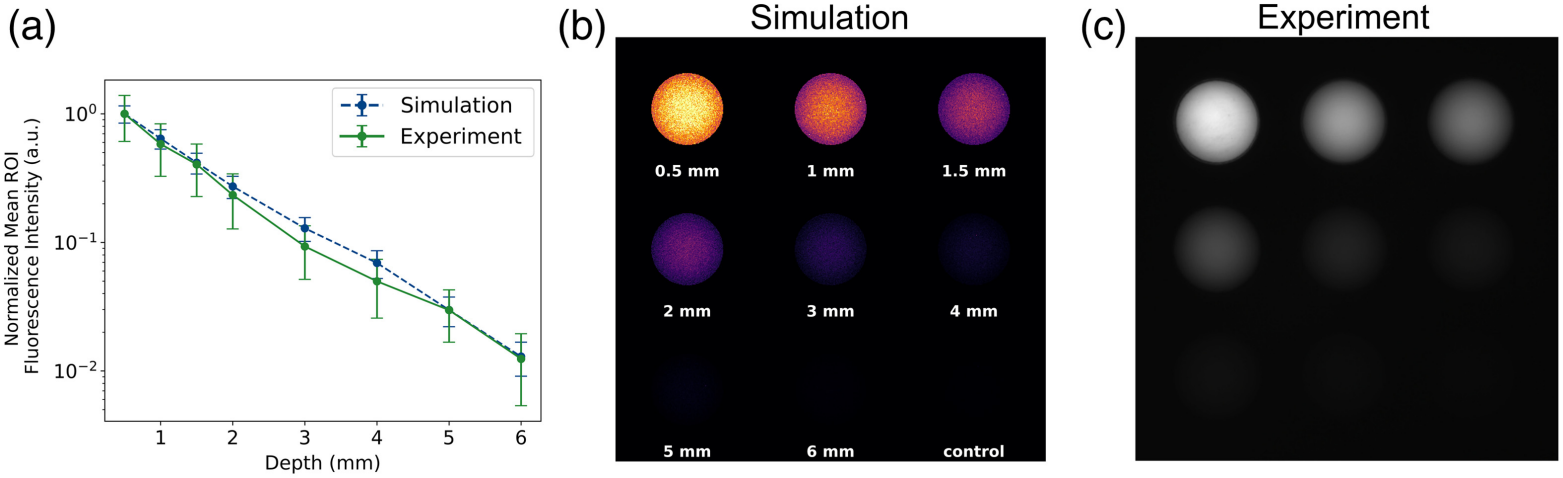
Results of the depth sensitivity target simulations and experiment. (a) Normalized mean ROI fluorescence intensity versus depth (0.5, 1, 1.5, 2, 3, 4, 5, and 6 mm) plot on a log-linear scale corresponding to the (b) MCX-ExEm simulated image and (c) experimental image of a QUEL Imaging ICG-equivalent depth sensitivity target. A 5 mm ROI (half-diameter) was taken for each circular well and normalized to the max value of the simulation or experiment values, respectively. The experiment values subtract the control well baseline before normalization (see Supplementary Material). Error bars represent the normalized standard deviation of each respective ROI (simulation and experiment).

Experimental fluorescence and MCX simulation results for an ICG-equivalent depth sensitivity target are shown in Fig. 6. The ROIs of the fluorescence images [Fig. 6(a)] show that there is good agreement between the simulations and the experimental phantom data, with a maximum normalized absolute error of 0.06, which is within the range of standard deviation values. Furthermore, the intensity exhibits a linear decrease on a log–linear scale as depth increases. Figures 6(b) and 6(c) visualize how closely simulation and experimental images match with this decrease in fluorescence signal over increasing depths. Each image was displayed using a linear look-up table to ensure comparable visualization of the data.

### 3.6 Depth Resolution Target (FluoFlow® Phantom)

**Fig. 7:**
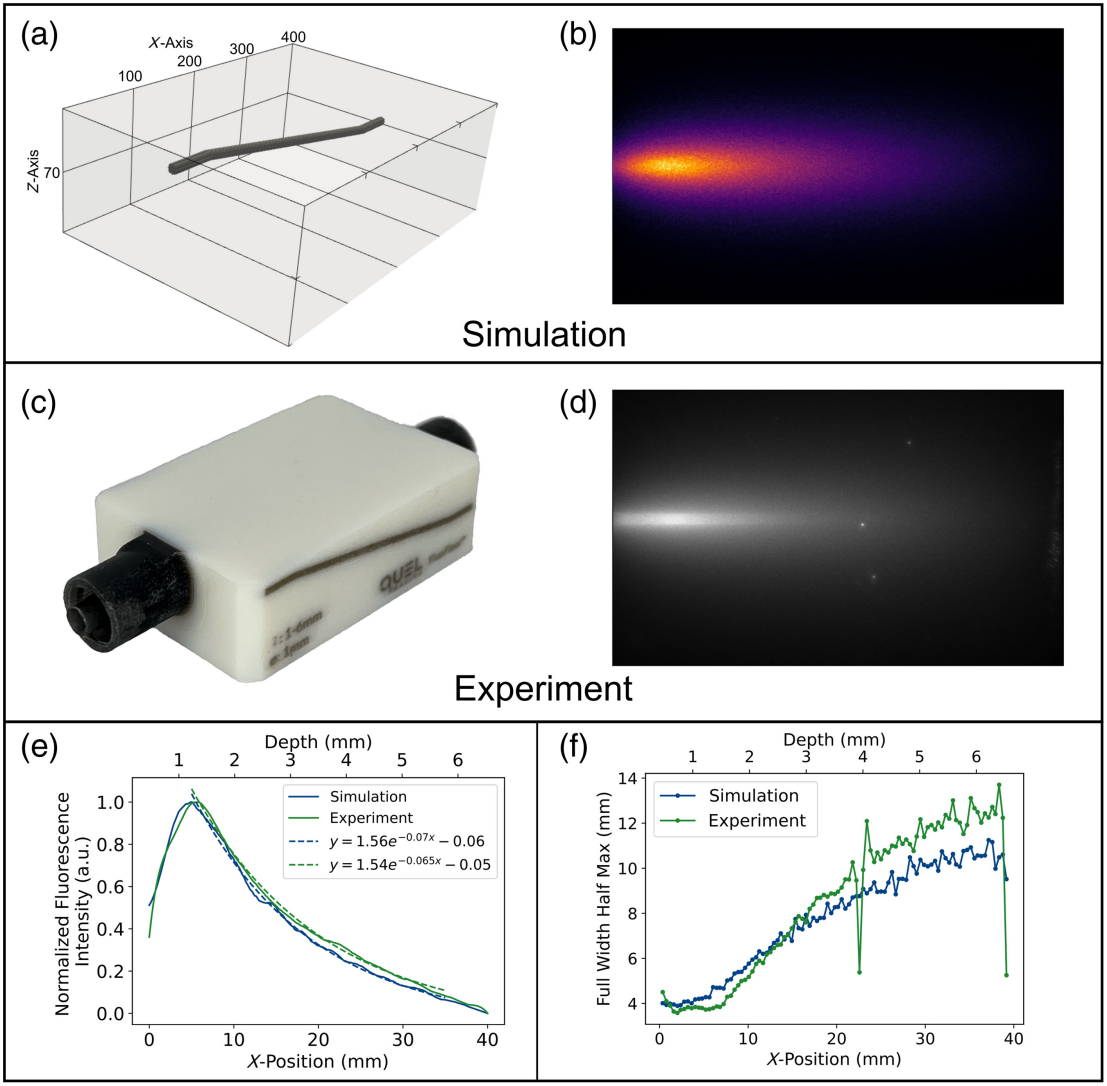
Depth resolution target (FluoFlow® phantom) with a varying depth channel (1 to 6 mm) is analyzed for the MCX-ExEm simulation and experiment. (a) MCX-ExEm simulation geometry, showing the 1 mm diameter channel structure. (b) Resulting simulated fluorescence images are compared with the physical phantom (c) and the experimental fluorescence image (d). The normalized fluorescence intensity along the center of the channel, fitted with exponential decay (e), and the full width at half maximum as a function of depth and x-position (f) is compared between the simulation and experiment.

Figure 7 shows the simulation and experimental results the FluoFlow® phantom, which is designed to assess depth resolution using an embedded channel that varies in depth. The simulation geometry is shown in Fig. 7(a), whereas the experimental phantom is shown in Fig. 7(c). The simulated fluorescence [Fig. 7(b)] and experimental [Fig. 7(d)] images both show the expected decrease in fluorescence intensity with increasing depth with a strong visual agreement between simulation and experiment. Each fluorescence image [Figs. 7(b) and 7(d)] was displayed using a linear look-up table to ensure comparable visualization of the data.

Figure 7(e) plots the fluorescence intensity through the centerline of the channel, revealing a sharp initial increase followed by an exponential decay in fluorescence intensity as depth increases. The experimental and simulated trends are well-aligned with an average normalized absolute error of 0.032, demonstrating that the MCX-ExEm model effectively captures fluorescence signal attenuation with depth for this embedded geometry.

To assess the lateral spread of fluorescence at different depths, FWHM values were extracted from orthogonal line profiles taken perpendicular to the channel at 100 evenly spaced x-positions. Figure 7(f) presents the FWHM values across the x-axis, showing that simulation and experimental results follow the same increasing trend, with FWHM widening at greater depths. However, a systematic mismatch is observed at deeper regions, where the experimental FWHM is consistently larger than the simulated FWHM. Several factors may contribute to this discrepancy, including uncertainties in measured optical properties, voxel resolution limitations in the simulation, and additional scattering effects in the experimental phantom that are not fully captured in the model. In addition, potential autofluorescence or slight misalignment in the experimental setup could lead to increased fluorescence spread at deeper locations.

## 4 Discussion

This study presents and validates MCX-ExEm, a voxel-based two-step Monte Carlo fluorescence simulation framework leveraging GPU acceleration. By utilizing MCX, an open-source, widely used Monte Carlo framework, these voxel-based fluorescence simulations can be easily adopted and extended by other researchers. Unlike previous studies that used liquid or gel phantoms with limited shelf lives and theoretically derived optical properties, this work integrates well-characterized solid phantoms with systematically measured optical properties. This approach provides a robust methodology for modeling fluorescence propagation in complex 3D geometries. The results demonstrate excellent agreement between MCX-ExEm simulations and experimental data across a diverse set of parameters, including variations in fluorophore concentration, bulk optical properties, object volume, and depth-dependent fluorescence attenuation.

The cuvette experiment results (Sec. 3.1) confirm that nonlinear fluorescence behavior, such as quenching effects at high ICG concentrations, can be predicted using MCX-ExEm simulations. The experimental and simulated data for ICG in a cuvette (Fig. 2) show that fluorescence intensity increases with concentration up to 3000 nM, after which a decline occurs due to reabsorption and inner filter effects.^45^ Importantly, MCX-ExEm captures this trend without requiring empirical correction factors, demonstrating the framework’s ability to simulate realistic fluorophore behavior. The simulated image data provide further insight into the fluorescent behavior than what the spectrofluorometer output provides. With simulations, the approximate concentration of quenching can be predicted, saving time rather than testing different fluorophore concentrations with serial dilutions.

Accurate simulation of fluorescence in biological tissue-mimicking materials requires careful consideration of bulk optical properties, including the reduced scattering and absorption coefficients. The results of the varying scattering and absorption phantoms (Fig. 3) confirm, as expected, that fluorescence emission is strongly influenced by these properties, and the agreement between MCX-ExEm simulations and experimental measurements supports the validity of the model in capturing these effects. The simulations captured key trends observed experimentally: fluorescence increased with higher scattering, likely due to the larger excitation energy deposited near the surface, whereas increasing absorption reduced overall fluorescence intensity due to attenuation of excitation light. The observed increase in fluorescence with higher scattering [Fig. 3(g)] suggests that enhanced light retention near the surface contributes to greater detected fluorescence. In contrast, higher absorption reduces fluorescence intensity [Fig. 3(h)] due to increased attenuation of excitation light before it reaches the fluorophore. These trends are consistent with established light transport principles in scattering-dominant media, indicating that MCX-ExEm accurately models fluorescence propagation under varying optical conditions. The largest discrepancy was observed for $\mu_{s}^{\prime }=0$, where experimental fluorescence intensity was lower than predicted by simulations. This difference is likely due to challenges in measuring optical properties at low scattering, as well as uncertainties in inverse adding-doubling estimates. Such differences highlight the importance of precise optical property characterization, particularly when using low-scattering materials. The validation of these phantoms shows that fluorescence can be well predicted across different optical properties and can be modified for any set of tissue-relevant optical properties.

The varying volume fluorescence experiments (Fig. 4) demonstrate that MCX-ExEm can quantitatively predict fluorescence intensity variations due to object size. To properly compare simulations for different-sized geometries, photon scaling needs to be applied in the second simulation step [Fig. 1(b)] to correct for differences in the photon generation of the 3D pattern source, where MCX scales the photons to be generated in the masked area. This means that if $10^{7}$ photons are used for the second step, the same $10^{7}$ photons would be distributed in a 2 mm diameter cylinder as in a 20 mm diameter cylinder when realistically the 2 mm diameter cylinder should not have the same amount of energy as the 20 mm diameter cylinder. Therefore, to accurately launch photons for the fluorescence simulation, the number of photons was scaled by the volume of each cylinder. Knowing the relative fluorescence intensities of objects of different sizes can help with the identification of different fluorescent objects such as tissues or tumors and also be useful in optimizing the sensitivity and resolution of imaging systems. Given the voxel-based framework of MCX, this scaling can be easily applied as part of the simulation workflow when comparing different geometries.

Standardized fluorescence reference targets are widely used to characterize imaging systems. As fluorescence-guided surgery imaging systems rapidly advance, the lack of standardization remains a significant challenge as described in depth in Pogue et al.^28^ Ochoa et al.^36^ demonstrated how the commercially available concentration sensitivity and depth sensitivity targets compare across different imaging systems, highlighting how these targets provide a solid foundation for testing and characterizing these systems. The concentration and depth sensitivity targets (Figs. 5 and 6) demonstrated strong agreement between simulations and experimental data, confirming that MCX-ExEm can serve as a computational extension of these reference standards. Physical phantoms can have fabrication limitations such as potential air gaps in multi-part phantoms such as the depth sensitivity target, which one might think would affect the fluorescence. However, simulations can demonstrate that effects such as these are non-significant as shown in Fig. S4 in the Supplementary Material. Similarly, fluorescence imaging systems have different camera specifications which can lead to varying angles of acceptance. We show in Fig. S5 in the Supplementary Material that the overall trend of the concentration sensitivity target is the same, no matter the acceptance angle. Ultimately, this proves the power of combining simulations and phantoms to ensure proper comparisons across imaging systems.

The FluoFlow® phantom experiment (Fig. 7) provided validation for a more complex geometry, assessing depth-dependent fluorescence attenuation and subsurface spatial resolution in an embedded channel structure. The simulated and experimental results showed good agreement in fluorescence intensity trends [Fig. 7(e)], with the expected exponential decay as depth increased through the center of the channel. The FWHM trends were also similar [Fig. 7(f)]; however, the simulation FWHM was lower than the experimental values at the larger depths, which be attributed to uncertainties in optical property measurements. With $\mu_{a}=0.0043\;{\mathrm{mm}}^{-1}$, this is in the low absorption regime where measuring optical properties is more difficult. To examine this further, the simulation was rerun with a $\mu_{a}=0.001\;{\mathrm{mm}}^{-1}$ (all other parameters fixed), and this showed closer agreement to the experiment FWHM values (see Fig. S6 in the Supplementary Material). This suggests that lower absorption values may introduce more noise due to experimental limitations of the optical property characterization. Another possible source of discrepancy is autofluorescence noise contribution from the background material. From a Monte Carlo simulation perspective, the results could potentially be refined by increasing the number of photons or voxels, as insufficient photon counts or coarse voxel resolution may contribute to underestimation in fluorescence spread. However, for larger and more complex geometries, a tradeoff exists among a number of photons, voxel size, computational memory, and simulation time. The same number of photons was used for this simulation as the other examples, but larger geometries could require more photons for greater accuracy.

Overall, the simulations showed very strong agreement with the experiments. Causes of errors are most likely attributed to optical property characterization, which highlights that simulations are only as good as the optical property characterization. Further advancement on uncertainty budgets with input parameters could better explore simulation errors. Future work includes phantom development of assorted geometries for different applications and making developments toward multispectral digital twins. With 3D printing, custom anthropomorphic phantoms can be easily manufactured and computationally modeled, allowing for the testing of a wide range of clinical applications. Multiple fluorophores can be tested with custom anthropomorphic phantoms as inclusions of varying shapes, positions, and fluorophores. On the modeling side, MCX-ExEm fluorescence can easily be modified for any fluorophore by measuring the absorbance and subsequently calculating the extinction and absorption coefficients at the excitation and emission wavelengths. The quantum yield can also be adjusted for various emission spectra. Simple modifications to the current framework for different fluorophores would allow for multispectral imaging applications. Other improvements to the model include adding experimental noise including autofluorescence, accounting for the emission of reabsorbed fluorophores, and time-resolved fluorescence to more accurately represent fluorescence imaging as a whole. Future studies addressing absolute fluorescence quantification through comprehensive characterization of excitation source power, detector response, and optical component properties would further enhance the applicability and robustness of MCX-ExEm modeling. These advancements will allow for more streamlined development and testing of fluorescence imaging systems.

## 5 Conclusions

This work presents a multi-parameter validated GPU-accelerated voxel-based Monte Carlo fluorescence simulation framework (MCX-ExEm), demonstrating its ability to predict fluorescence intensity distributions across a range of experimental conditions. By systematically incorporating measured optical properties and well-characterized solid phantoms, this approach provides a validated method for fluorescence simulations in structured 3D geometries. MCX-ExEm accurately replicates fluorescence phenomena, capturing quenching at high fluorophore concentrations and variations driven by bulk scattering and absorption. It reliably predicts intensity scaling with volume, underscoring the need for proper photon scaling in voxel-based simulations. Validation with structured phantoms—addressing both concentration and depth sensitivities—shows strong agreement between simulated and experimental images. Depth-dependent fluorescence attenuation was demonstrated for an embedded channel geometry, signifying the ability to capture more complex geometries through 3D-printed phantoms and simulations. Integrating simulation results with validated physical phantoms establishes a robust digital twin framework that enables virtual testing and iterative refinement of imaging systems with the ability to customize various parameters.

## Supplementary Material

10.1117/1.JBO.30.S3. S34104.s01

## Data Availability

Additional figures and tables are available in the Supplementary Material. The data that support the findings of this study are available from the corresponding author upon reasonable request. The simulation input files, configurations, and data used in this study are publicly available at https://github.com/QUEL-Imaging/MCX-ExEm
