# Supplementary material for: Toward fluorescence digital twins: multi-parameter experimental validation of fluorescence Monte Carlo simulations using solid phantoms
Source: J Biomed Opt. 2025 May 27;30(Suppl 3):S34104. doi: 10.1117/1.JBO.30.S3.S34104 (PMC12119851; doi:10.1117/1.JBO.30.S3.S34104)
Supplement: Supplementary file 1 [file JBO_030_S34104_SD001.pdf]

Supplementary Material:

Towards Fluorescence Digital Twins: Multi-parameter Experimental Validation of Fluorescence Monte Carlo Simulations using Solid Phantoms

Mayna H. Nguyen,[a*] Ethan P. M. LaRochelle,[a] Edwin A. Robledo,[a] Alberto J. Ruiz[a]

[a]QUEL Imaging, 85 N Main St. St #142, White River Junction, Vermont, USA

1000 nM Concentration ICG Well									
**N photons**	**5 mm ROI mean**	**5 mm ROI stdev**	**5 mm ROI mean / N photons**	**ROI % change from 10^9 photons**	**5 mm ROI stdev / N photons**	**Step 1 time (s)**	**Step 2 time (s)**	**Step 1 photon / ms**	**Step 2 photon / ms**
1.00E+03	4.07E-08	3.91E-07	4.07E-11	834.780	3.91E-10	0.87	0.83	200	200
1.00E+04	7.99E-08	2.77E-07	7.99E-12	83.700	2.77E-11	1.09	1.19	1666.67	2000
1.00E+05	4.93E-07	6.22E-07	4.93E-12	13.366	6.22E-12	1.20	1.31	9090.91	7692.31
1.00E+06	4.38E-06	1.85E-06	4.38E-12	0.615	1.85E-12	1.28	1.33	16129.03	12500
1.00E+07	4.35E-05	5.96E-06	4.35E-12	0.040	5.96E-13	1.78	2.02	18484.29	12953.37
1.00E+08	4.35E-04	2.17E-05	4.35E-12	0.003	2.17E-13	6.49	9.00	18978.93	12845.22
1.00E+09	4.35E-03	1.29E-04	4.35E-12	0.000	1.29E-13	53.67	79.22	19059.24	12819.53

Table S1. Table of ROI mean values, ROI standard deviations, and simulation times in seconds and photon/ms vs number of photons for a 1000 nM concentration ICG well.

To determine the convergence, the mean ROI value is divided by N photons because when modeling fluorescence, as N photons increase, more fluorescence photons are generated and therefore the ROI values scale with N photons.

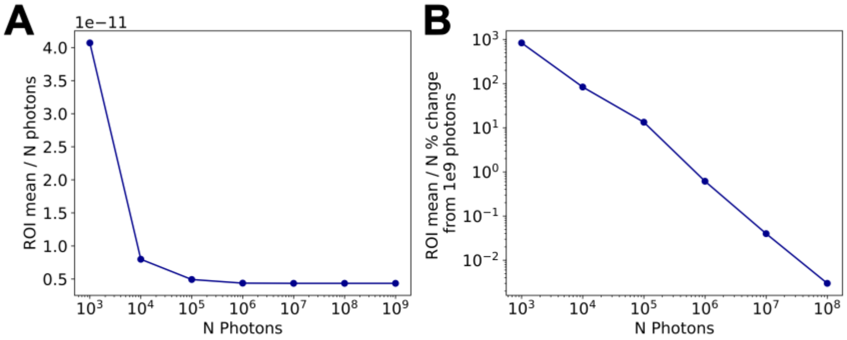

Figure S1. (A) The scaled ROI mean value as a function of N photons and (B) the % change of the scaled ROI mean value from the 10^9 scaled ROI mean value on a log scale.

**Depth Resolution Phantom**				
**Voxelization time (s) - not dependent on N photons: 146.885318918**				
**N Photons**	**Step 1 Time (s)**	**Step 2 Time (s)**	**Step 1 photon / ms**	**Step 2 photon / ms**
1.00E+03	1.59	1.89	45.45	9.8
1.00E+04	2.62	3.05	416.67	270.27
1.00E+05	3.30	3.52	2000	1176.47
1.00E+06	3.70	4.11	3846.15	1461.99
1.00E+07	6.23	9.89	3652.3	1523.93
1.00E+08	32.70	67.31	3424.77	1566.32
1.00E+09	300.30	638.03	3369.08	1575.66

Table S2. Table of simulation time in seconds and photon/ms vs number of photons for each simulation step of the depth resolution phantom.

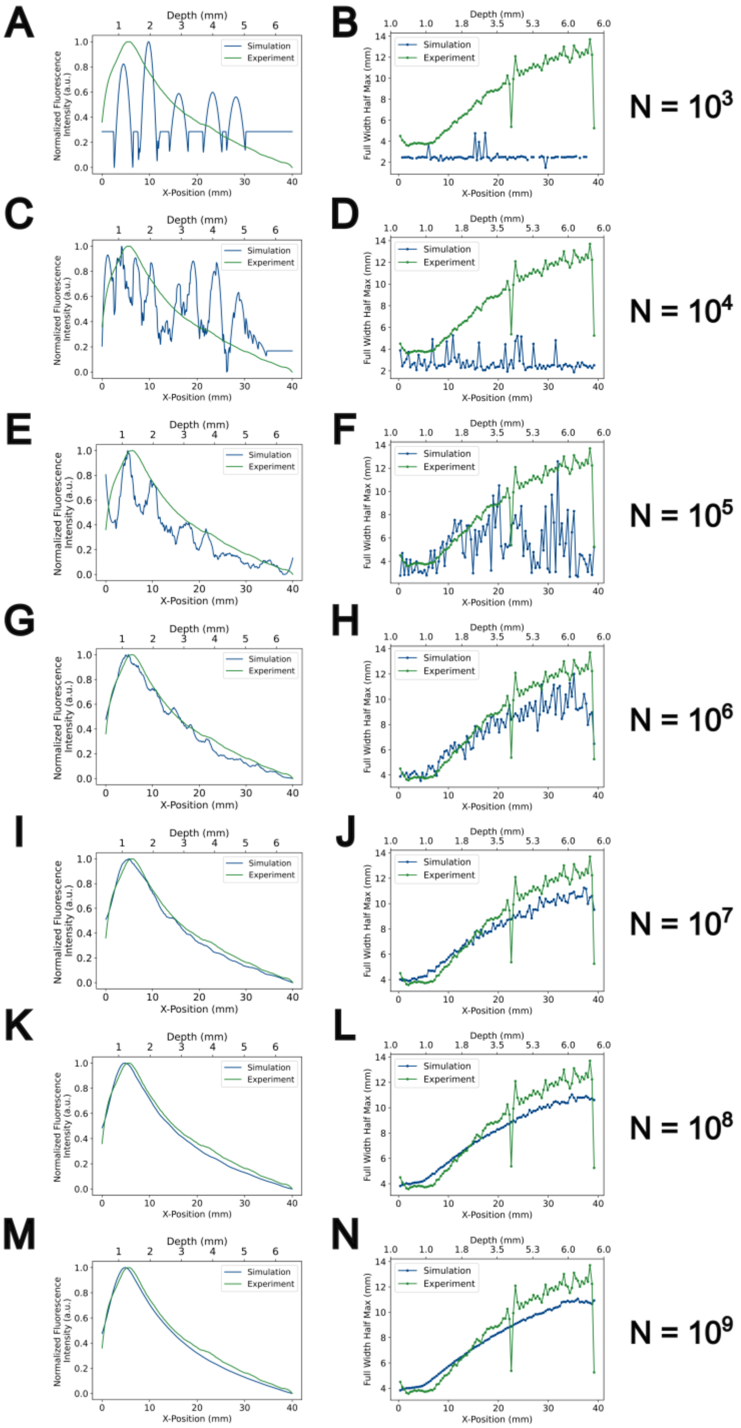

Figure S2. (A, C, E, G, I, K, M) Normalized fluorescence intensity along the center of the channel and the full width at half maximum (FWHM) (B, D, F, H, J, N) as a function of depth and x-position is compared between the simulation and experiment for a depth resolution FluoFlow® phantom with varying photons ($N = 10^3 – 10^7$).

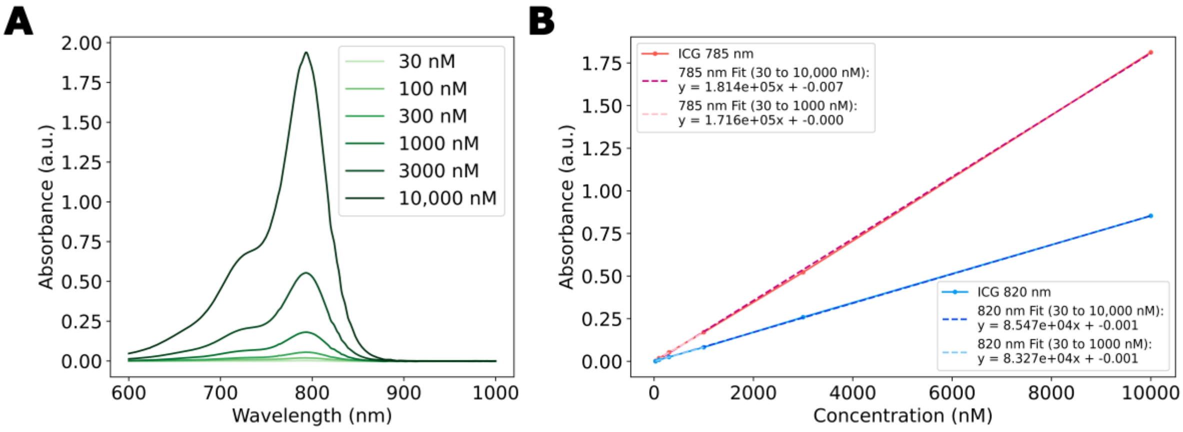

Figure S3. (A) Absorbance spectra of ICG in DMSO for concentrations 30 – 10,000 nM. (B) ICG absorbance vs concentration at 785 nm (red) and 820 nm (blue) with dotted fit lines.

Diameter (mm)	Height (mm)	Volume (mm^3)
2	1.33	4.19
4	2.67	33.51
8	5.33	268.07
10	6.67	523.60
15	10.00	1767.15
20	13.33	4188.79

Table S3: Table of cylinder dimensions for varying volumes.

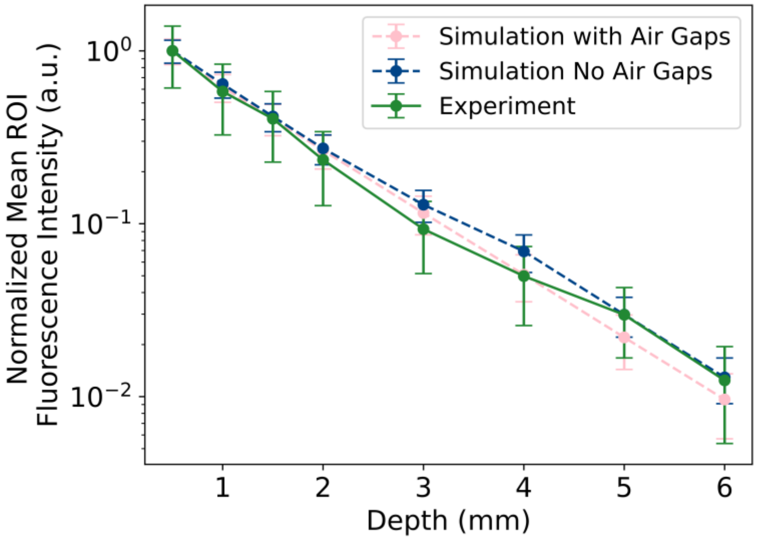

Figure S4. Results of the depth sensitivity target simulations with air gaps, simulations without air gaps, and experiment. Normalized mean ROI fluorescence intensity vs depth (0.5, 1, 1.5, 2, 3, 4, 5, 6 mm) plot on a log-linear scale corresponding to the MCX-ExEm simulated images with a 200 μm air gap between the top layer and bottom fluorescence layer, MCX-ExEm simulated images with no air gap, and experimental image of a QUEL Imaging ICG-equivalent depth sensitivity target. A 5 mm ROI (half-diameter) was taken for each circular well and normalized to the max value of the simulation or experiment values, respectively. The experiment values subtract the control well baseline before normalization (see supplemental data). Error bars represent the normalized standard deviation of each respective ROI (simulation and experiment).

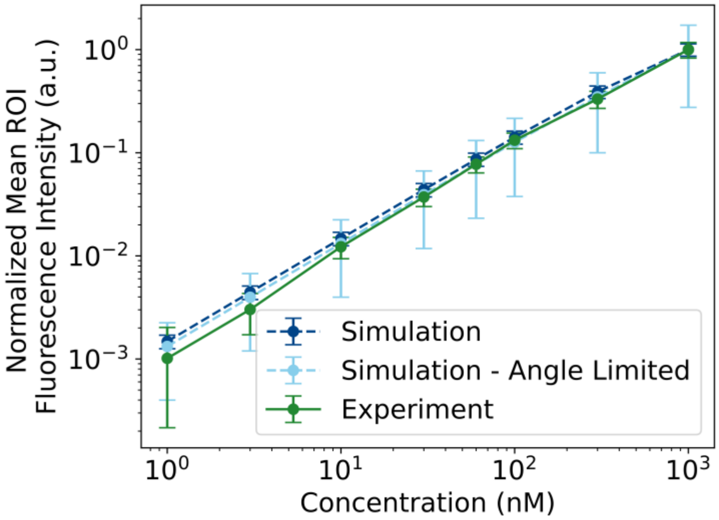

Figure S5. Results of the concentration sensitivity target simulations with diffuse reflectance detector, simulations with 10° angle limited detector, and experiment. Normalized mean ROI fluorescence intensity vs concentration (1, 3, 10, 60, 100, 300, 1000 nM) on a log-log scale for MCX-ExEm simulations and experiments corresponding to the MCX Ex-Em simulation images and experimental fluorescence image of a QUEL Imaging ICG-equivalent concentration sensitivity target. A 5 mm ROI (half-diameter) was taken from each circular well and normalized to the max value of the simulation or experiment values, respectively. The experiment values subtract the control well baseline before normalization (see supplemental data). Error bars represent the normalized standard deviation of each respective ROI (simulation and experiment).

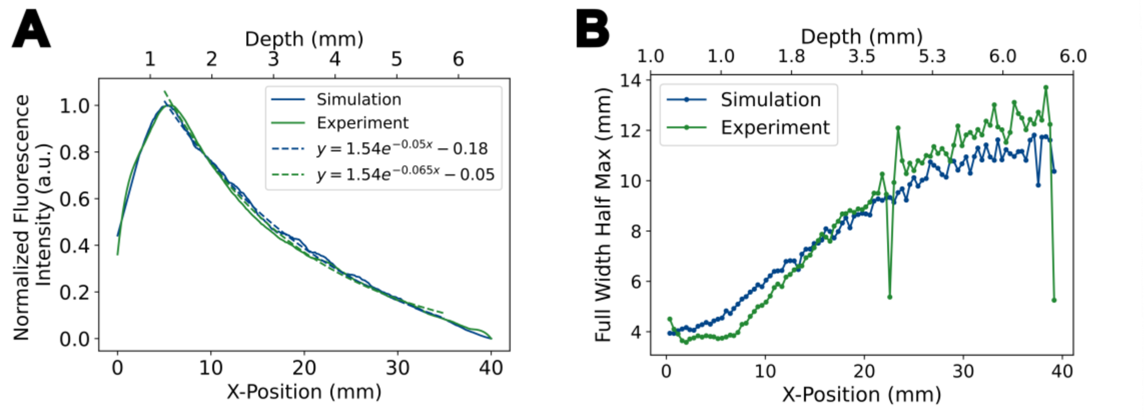

Figure S6. (A) Normalized fluorescence intensity along the center of the channel fitted with exponential decay and the full width at half maximum (FWHM) (B) as a function of depth and x-position is compared between the simulation and experiment for a depth resolution FluoFlow® phantom with base material $\mu_a = 0.001$ mm^{-1}, all other parameters fixed.
